# Electroporation by subnanosecond pulses

**DOI:** 10.1016/j.bbrep.2016.05.002

**Published:** 2016-05-03

**Authors:** Iurii Semenov, Shu Xiao, Andrei G. Pakhomov

**Affiliations:** a Frank Reidy Research Center for Bioelectrics, Old Dominion University, Norfolk, VA, USA; b Department of Electrical and Computer Engineering, Old Dominion University, Norfolk, VA, USA

**Keywords:** EP, electric pulses, MP, membrane potential, nsEP, nanosecond electric pulses, psEP, picosecond electric pulses, VGCC, voltage-gated calcium channels, RHN, rat hippocampal neurons, Picosecond pulses, Electroporation, Electropermeabilization, Nanopores, Patch clamp, Membrane permeability

## Abstract

Electropermeabilization of cell membranes by micro- and nanosecond-duration stimuli has been studied extensively, whereas effects of picosecond electric pulses (psEP) remain essentially unexplored. We utilized whole-cell patch clamp and Di-8-ANEPPS voltage-sensitive dye measurements to characterize plasma membrane effects of 500 ps stimuli in rat hippocampal neurons (RHN), NG108, and CHO cells. Even a single 500-ps pulse at 190 kV/cm increased membrane conductance and depolarized cells. These effects were augmented by applying brief psEP bursts (5–125 pulses), whereas the rate of pulse delivery (8 Hz–1 kHz) played little role. psEP-treated cells displayed large inward current at negative membrane potentials but modest or no conductance changes at positive potentials. A 1-kHz burst of 25 pulses increased the whole-cell conductance in the range (−100)–(−60) mV to 22–26 nS in RHN and NG108 cells (from 3 and 0.7 nS, respectively), but only to 5 nS in CHO (from 0.3 nS). The conductance increase was reversible within about 2 min. Such pattern of cell permeabilization, with characteristic inward rectification and slow recovery, was similar to earlier reported effects of 60- and 600-ns pulses, pointing to the similarity of structural membrane rearrangements in spite of a different membrane charging mechanism.

## Highlights

• 500-ps pulses at 190 kV/cm electroporate mammalian cells.
• Cells lose membrane potential and increase conductance at negative potentials.
• Permeabilization is enhanced by increasing pulse number but not the rate.
• Membrane resealing takes minutes.

## 1 Introduction

Application of high-voltage electric pulses (EP) of micro- or millisecond duration to living cells is a well-established technique to increase cell membrane permeability and introduce normally impermeable substances into cells [1], [2], [3]. This process, termed electroporation or electropermeabilization, has numerous applications in experimental biology, medicine, and biotechnology.

In parallel with the advancement of pulsed power engineering during the last decades, electroporation research expanded to shorter and higher amplitude electric pulses. Early work with nanosecond EP (nsEP) focused on electroporation of intracellular membranes, whereas the plasma membrane was thought to stay unaffected [4], [5], [6], [7]. Later theoretical and experimental studies established that nsEP cause the formation of long-lived nanopores in the plasma membrane [8], [9], [10], [11]. These nanopores had complex conductive properties, including voltage and current sensitivity, inward rectification, and ion selectivity. Interestingly, similar features could occasionally be established in cells permeabilized by ms-range EP or even subjected to hyperpolarization in a low K^+^ solution [12], [13], [14]. Nanopores could supposedly be created by “long” EP either as a fraction of a mixed-size pore population, or by shrinking of larger electropores. Gd^3+^ and La^3+^ ions were effective at inhibiting or preventing pore conductance regardless of the exact permeabilization method [10], [12], [14], [15], [16]. Although the data suggest similar properties of nanopores produced by different treatments, this conjecture has yet to be tested by direct experiments.

Furthermore, our recent data on Ca^2+^ mobilization by picosecond-range EP (psEP) [17] could not be easily explained by existing paradigms of electropore formation. We established that Ca^2+^ activation by 500-ps, 190 kV/cm EP is mediated by opening of voltage-gated calcium channels (VGCC) and therefore does not occur in CHO cells (which do not express any VGCC). This observation contrasted numerous findings that 60-ns and longer EP do not rely on VGCC and efficiently activate Ca^2+^ in CHO cells [18], [19], [20], [21]. Therefore, findings with psEP could be indicative of a non-conventional membrane electroporation, with pores so short-lived that usual methods of pore detection fail. One may also speculate that the lack of VGCC expression or some unknown differences in membrane composition made CHO cells less vulnerable to psEP, although it was not the case for nsEP (CHO cells showed similar sensitivity to 60- and 600-ns pulses as VGCC-expressing NG108 and GH3 cells [22], [23]). Finally, one may think of VGCC activation by psEP in the absence of electroporation, just by psEP-induced depolarization of the plasma membrane, similarly to conventional electrostimulation. However, 500-ps pulses are too short to change the membrane potential by Maxwell-Wagner polarization and have to rely on the dielectric stacking effect instead. As a result, the relaxation of the psEP-induced membrane potential is essentially instant and happens roughly 1000 times faster than movement of the voltage sensor of a VGCC; hence, direct VGCC activation by psEP not mediated by electroporation is also difficult to explain [17].

The present work was aimed at further exploring psEP-induced electroporation by electrophysiological and optical membrane potential detection techniques. Bioeffects of psEP remain essentially an uncharted territory, with current knowledge limited to a few isolated reports [17], [24], [25], [26]. Below we demonstrate that even a single psEP at 190 kV/cm can permeabilize cell membrane, and that psEP- and nsEP-porated membranes share similar features. At the same time, CHO cells proved to be less sensitive to psEP than other tested cells, which contrasts earlier findings using nsEP [22], [23] but is consistent with the lack of Ca^2+^ activation by psEP [17].

## 2 Materials and methods

### 2.1 Cells and media

Chinese hamster ovary cells CHO-K1 and a murine neuroblastoma-rat glioma hybrid NG108 were obtained from the American Type Culture Collection (ATCC, Manassas, VA). They were propagated at 37 °C with 5% CO_2_ in air according to the supplier's recommendations. CHO cells were grown in Ham’s F12K medium (Mediatech Cellgro, Herdon, VA) supplemented with 10% fetal bovine serum (FBS), 100 I. U./ml penicillin and 0.1 μg/ml streptomycin. NG108 cells were cultured in Dulbecco's Modified Eagle's medium (Caisson Labs, North Logan, UT) without sodium pyruvate, supplemented with 4 mM l-glutamine, 4.5 g/L glucose, 10% FBS, 0.2 mM hypoxanthine, 400 nM aminopterin, and 0.016 mM thymidine (without antibiotics). The media supplements were from Sigma-Aldrich (St. Louis, MO) except for the serum (Atlanta Biologicals, Norcross, GA). For the passage immediately preceding experiments, cells were transferred onto glass coverslips. Cells were used in experiments after 12–24 h of growing on the coverslips.

Dissociated E18 rat hippocampal neurons (RHN) were purchased from BrainBits LLC (Springfield, IL) and seeded on poly-d-lysine/laminin coated glass coverslips (Corning, Corning, NY) in Gibco Neurobasal medium supplemented with 50× B-27 (20 ml/l) and 100× Glutamax (2.4 ml/l) (all from Thermo Fisher Scientific, Waltham, MA). One half of the medium was replaced every 3 days. Neurons were used between 1 and 3 weeks in culture.

### 2.2 Electrophysiology

Whole-cell mode of conventional voltage clamp or current clamp (at I=0) were used to quantify psEP-induced changes in the membrane conductance and resting membrane potential (MP), respectively. The measurements were performed using Axopatch 200B amplifier, Digidata 1440 A board, and Clampex v. 10.2 software (Molecular Devices, Sunnyvale, CA). Coverslips with cells were placed in a glass-bottomed perfusion chamber (Warner Instruments, Hamden, CT) mounted on a stage of an IX71 microscope (Olympus America, Center Valley, PA). The extracellular solution contained (mM): 140 NaCl, 5 KCl, 2 CaCl_2_, 1.5 MgCl_2_, 10 HEPES, and 10 glucose (pH 7.2). Recording pipettes were manufactured by pulling borosilicate glass (BF-150–866–10, Sutter Instrument, Novato, CA) to a tip resistance of 1–3 MOhm using a Flaming/Brown P-97 Micropipette puller (Sutter, Novato, CA), and filled with (mM): 10 NaCl, 130 KCl, 2 CaCl_2_, 3 MgCl_2_, 10 HEPES, and 5 K-EGTA (pH of 7.2). All chemicals were purchased from Sigma-Aldrich (St. Louis, MO).

The membrane conductance measurement protocols and procedures were similar to described previously [8], [9], [15], [27]. The positioning of the recording pipette with respect to psEP-delivering electrodes was the same as illustrated in [23], with the exception of the fact that the gap between the psEP-delivering electrodes in the current study was somewhat different. Within 1–2 min after the whole-cell configuration was established, membrane currents were measured by applying a voltage-step protocol (80-ms steps from −100 to 40 mV in 10-mV increments) from the holding potential of −80 mV. This protocol took about 3 s, and it was applied at 5 s before psEP exposure and again at 1, 5, 30, and 120 s after it. The current at each step was measured after the steady-state level was reached, i. e., at 30–50 ms into the step. Thus, we generated a series of current-voltage (I-V) curves to compare the whole-cell currents before psEP exposure and at indicated time intervals after it. The whole-cell conductance was measured by linear fitting of the I-V curves in individual cells in the range from −100 to −60 mV.

The membrane voltages reported in this paper have not been corrected for the junction potential (which equaled 4.2 mV).

### 2.3 Optical membrane potential monitoring with Di-8-ANEPPS

To load Di-8-ANEPPS into cell plasma membrane, coverslips with cells were incubated in the extracellular solution with 20 μM of the dye for 45 min at 4 °C. The coverslips were transferred into the glass-bottomed perfusion chamber and the excess dye was rinsed off.

The MP was measured by ratiometric imaging, which enabled a more reliable calibration, better signal-to-noise ratio, and reduced the impact of bleaching. The dye was excited alternately in 5-ms windows at 440 and 530 nm using fast wavelength switcher Lambda DG4 (Sutter Instruments, Novato, CA). We utilized a U-N71006 Di-8-ANEPPS filter set (Chroma Technology, Bellows Falls, VT) and a PlanApo N 60×/1.42 objective (Olympus). Emission was measured at 605 nm with an iXon Ultra 897 back-illuminated CCD Camera (Andor Technology, Belfast, UK). Dye emission ratio was calibrated against the membrane potential in voltage-clamped NG108 cells.

MP measurements with the dye typically began 2 s prior to psEP delivery and continued for 10 s after the exposure, at 100 image pairs/s. The image acquisition and on-line data analysis were accomplished with Metafluor v.7.5. (Molecular Devices). An FFT filter utility of Origin 8.0 (OriginLab Corporation, Northampton, MA) was employed for offline noise reduction.

### 2.4 psEP exposure and dosimetry

We utilized the same psEP delivery and measurement techniques as reported recently [17]. In brief, pulses were produced by an FPG 20–1 PM generator (FID GmbH, Burbach, Germany). They were triggered externally and synchronized with image acquisitions or voltage step protocols using Digidata 1440 A board. The exact timing of psEP delivery, pulse rate, and the number of pulses were all programmed in Clampex software.

The pulses were sent to a 4 GHz, 20 Gs/s TDS7404 oscilloscope (Tektronix, Beaverton, OR) and to a π-network intended to absorb reflections from the load. psEP were delivered to cells in the bath by means of a pair of tungsten rods (100 µm diameter, 170 µm gap) at the end of a 50-Ω RG316 coaxial cable. The electrode assembly was driven by an MPC-200 robotic manipulator (Sutter Instruments, Novato, CA), to place the tips of the rods precisely at 50 µm above the coverslip surface with the selected cell(s) being in the middle of the gap between them.

The electric field values at the location of cells were determined by simulation with a 3D time-domain electromagnetic solver, CST microwave studio (Framingham, MA). The simulation also established broadening of the electric pulse due to the impedance mismatch, from 320 ps at the generator output to approximately 500 ps at the load (as measured at 50% of the peak amplitude). The simulation did not include possible electric field distortion by the exposed cell itself or by the patch clamp pipette.

## 3 Results and discussion

### 3.1 Changes in the whole-cell conductance after a burst of 25 EP (500 ps, 190 kV/cm, 1 kHz)

**Fig. 1:**
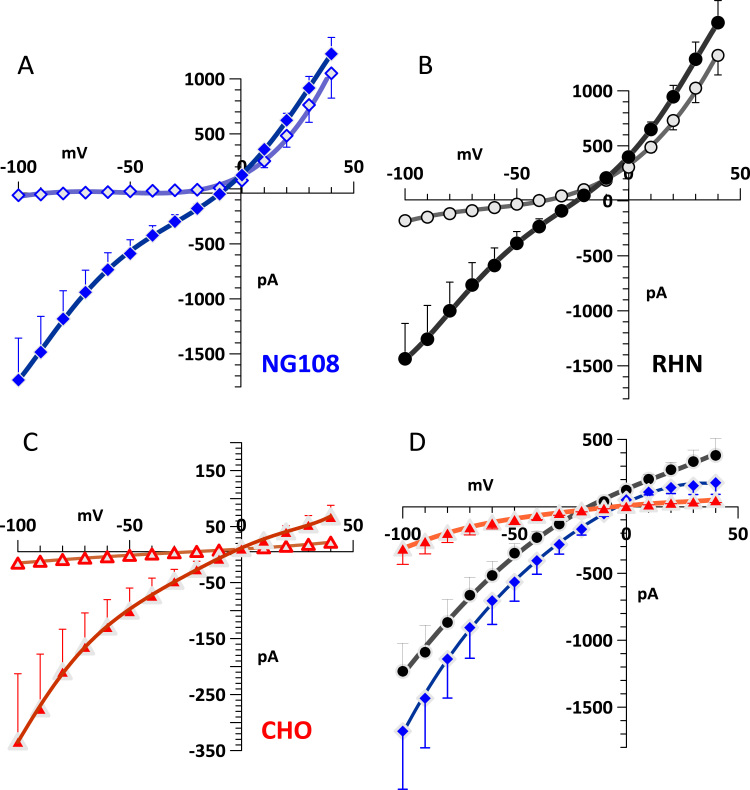
Change in whole-cell currents by a burst of 25, 500-ps pulses (190 kV/cm, 1 kHz). (A-C) Current-voltage curves for NG108, RHN, and CHO cells recorded immediately prior to psEP (open symbols) and after it (filled symbols; the voltage step protocol started 1 s after psEP and took 3 s). Mean±s.e. for 8–10 cells in each group; for clarity, error bars are shown in one direction. Note a different vertical scale in panel C (CHO cells). Sham exposures caused no appreciable change in currents (data not shown). (D) Currents due to the new conductance pathway created by psEP in the same three cell lines. Each curve was obtained by subtracting pre-psEP currents from post-psEP currents in individual cells, followed by averaging the data across the group. See text for more details.

These treatment parameters were chosen because they caused consistent, reproducible, and long-lasting but reversible increase of whole-cell currents in all studied cell types (Fig. 1, A-C). At the same time, the total energy delivered by such trains was low and heating by psEP, even under the “worst case scenario” (adiabatic) conditions did not exceed 1.5 °C [17]. The current-voltage (I-V) curves measured by a step protocol that was initiated at 1 s after psEP delivery were distinguished by a strong increase of inward current at negative transmembrane potentials, but a modest, if any, increase of the outward current at positive potentials. Moreover, the I-V curves became steeper as the membrane potential became more negative, reflecting the voltage sensitivity of the inward conductance. These I-V curve features were remarkably similar to earlier findings with much longer 60- and 600-ns pulses [8], [9], [23], [28], [29]. The inward rectification along with voltage sensitivity were regarded as electrophysiological hallmarks of nanopore formation and distinguished them from linear I-V dependence of larger membrane pores [8], [9]. Both the inward rectification and voltage sensitivity could potentially be explained by a conical, asymmetric shape of nanopores [8], or by nanopore opening at the bottom of funnel-shaped membrane invaginations, such as caveoles, but this hypothesis has not been experimentally verified.

Notably, psEP increased the conductance to a much greater extent in RHN and NG108 cells than in CHO cells. Specifically, the conductance measured by a linear fit of I-V curves in the range from −100 to −60 mV increased, on average, to 21±2.3 nS in RHN (from 3±0.34 nS); to 23±5.2 nS in NG108 (from 0.71±0.24 nS); and to only 4.7±0.8 nS in CHO cells (from 0.28±0.04 nS). The difference between post- and pre-exposure I-V curves is presumably the current carried through psEP-opened membrane pores (Fig. 1(D)). Opening of some endogenous ion channels by psEP could potentially contribute to this current, although previous studies with nsEP did not identify any specific channels involved [8], [9], [10], [30]. Smaller values of “psEP-added current” in CHO cells could reflect less efficient opening of pores (e. g., because of a different membrane composition [31], [32], [33]) or a lower expression of an unidentified endogenous ion channel that is activated by psEP). The fact that in earlier studies [22], [23] permeabilization of CHO cells by 60- and 600-ns EP was no less efficient than of GH3 or NG108 is an argument against involvement of unidentified ion channels, although the efficiency criteria used in these studies were somewhat different and the comparison is not fully valid.

**Fig. 2:**
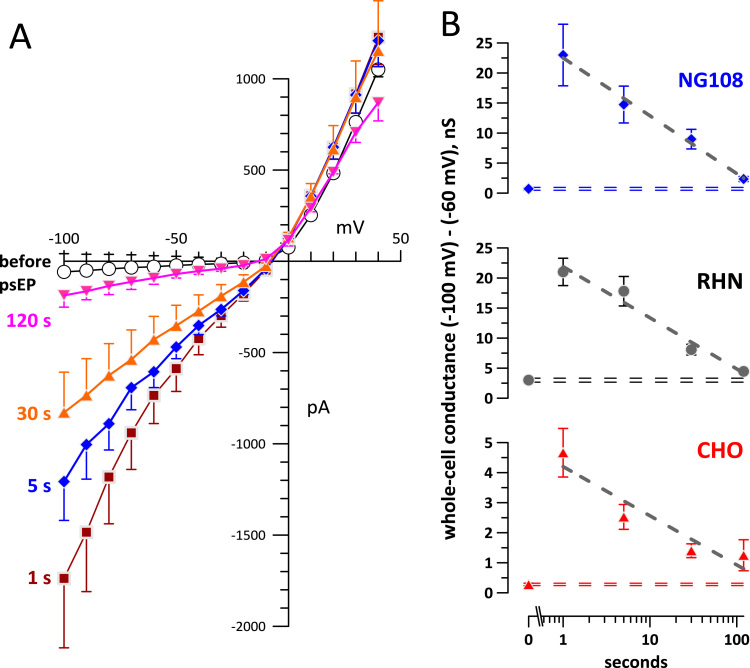
Membrane resealing as reflected by the gradual reduction of membrane conductance with time after exposure to 25 psEP (190 kV/cm, 1 kHz). (A) Current-voltage curves in NG108 cells before psEP (opens symbols) and at indicated timepoints after it (mean±s.e., n=8). (B) The time course of the membrane conductance decrease within 2 min after psEP, in 3 different cell types. The conductance was measured by linear fitting of the current-voltage curves in individual cells in the range from −100 to −60 mV (mean±s.e., n=8–10). Dashed horizontal lines denote the range of conductance values (mean±s.e.) prior to psEP exposure. Note a different vertical scale for CHO cells. See Fig. 1 and text for more details.

The restoration of the initial membrane conductance was gradual and took minutes (Fig. 2). The time course of membrane recovery was similar in different cell types, despite different starting values (Fig. 2(B)). This time course of resealing was similar to what was reported in studies with nsEP [8], [9], [28], once again pointing to the similarity of pore properties.

### 3.2 Effects of smaller psEP amplitude and of single pulses

Exposure of living cells to high-amplitude psEP remains a challenging cutting-edge technology, and currently we do not have the flexibility to gradually adjust the pulse amplitude without altering pulse shape or duration. Instead, we could use an in-line fixed attenuator to bring the psEP amplitude down to 76 kV/cm. However, at this amplitude we could not elicit any psEP effects, regardless of the number of pulses delivered (data not shown).

When using a single 190 kV/cm psEP instead of 25-pulse bursts, we observed a modest but statistically significant conductance increase at negative MP in NG108 cells (from 0.73±0.13 nS to 1.5±0.17 ns, n=10, p<0.05), but no consistent effect in either RHN or CHO cells. Within the accuracy of the methods employed, the value of 190 kV/cm can be considered to be at or near the threshold of membrane permeabilization by 500-ps EP.

### 3.3 The loss of the resting MP due to permeabilization by psEP: comparison of patch clamp and optical MP monitoring

While patch clamp is a powerful method to study cell membrane electropermeabilization, it has to be used with caution due to the inherent possibility of artifacts due to nsEP or psEP pick-up by the patch clamp amplifier. In early studies, the recording pipette was kept away from the cell and the whole cell configuration was not attempted until after the pulse treatment [10], [30]. Later on, we found that already “patched” cells responded to nsEP similarly to intact cells and corroborated this observation by concurrent fluorescent dye uptake measurements [9], [27], [28], [34]. Nonetheless, much higher amplitude of psEP used in this study (compared to more common 5–15 kV/cm for nsEP) required additional proof of the method.

Instead of dye uptake, we chose to compare MP values as measured by current clamp (in I=0 mode) and by an MP-sensitive fluorescent dye, Di-8-ANEPPS. The loss of resting MP is a well-established consequence of cell permeabilization by nsEP [30], hence a similar impact of permeabilization by psEP was expected. These experiments were performed in NG108 cells only.

**Fig. 3:**
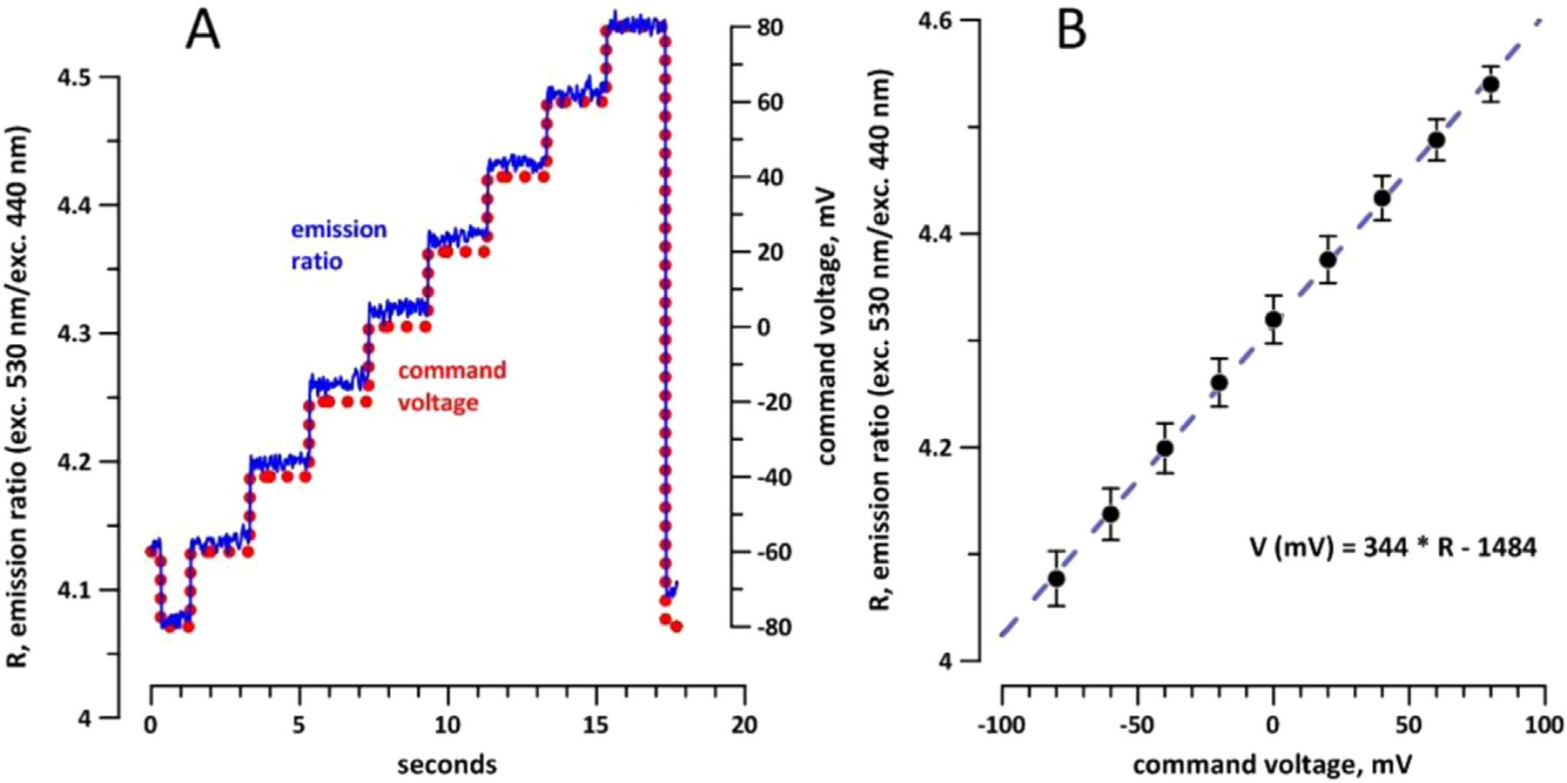
Calibration of Di-8-ANEPPS dye for membrane potential measurements using whole-cell patch clamp. The dye response was characterized by a ratio (R) of emission intensities when the dye was alternately excited at 530 and 440 nm. (A) Voltage steps imposed by a whole-cell voltage clamp protocol (dotted line, right scale) and respective changes in R (solid line, left scale) in a representative NG108 cell. (B) A liner dependence of R on the membrane potential in a single batch of dye-loaded cells (mean values±s.e., n=5). The linear equation enables calculation of the membrane potential from R values, although the intercept term varies between different cell batches. See text for more details.

As a first step, the dye emission was calibrated against MP as controlled by voltage clamp (Fig. 3(A)); no psEP were applied. The dye response was measured as fluorescence ratio at two excitation wavelengths, as described above in Methods. In different individual cells, the dye response depended linearly on the membrane voltage, with good reproducibility between different cells of the same batch (Fig. 3(B)). For different cell batches, the slope coefficient of the linear equation remained the same but the intercept term varied. Therefore the method could be utilized to measure MP changes (∆MP) but not the absolute MP values.

**Fig. 4:**
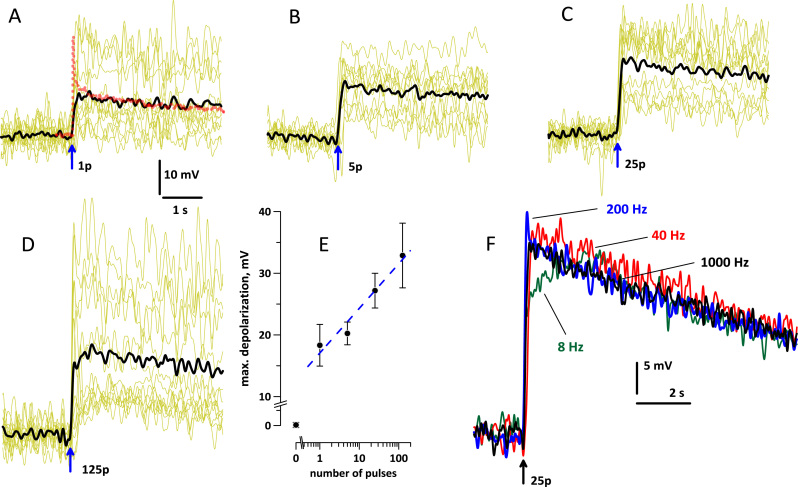
Effect of psEP stimulation on the resting membrane potential (MP) in NG108 cells. (A-D) Changes in the optical MP evoked by 1, 5, 25, and 125 pulses (arrow) at 190 kV/cm, 1 kHz, 500-ps pulse duration. Shown are representative traces in individual cells (yellow lines, 11–13 cells per plot) and their average (black). In (A), the dotted red line is MP change as measured by whole-cell patch clamp (the average of 11 cells). The peak at the moment of psEP application is an electrical pick-up artifact (truncated). (E) The maximum change of the membrane potential within 1 s after the stimulation, averaged for the experiments in panels (A-D) and also for sham exposures (0 pulses). Mean±s.e., n=11–13; dashed line is the best fit using log function. (F) Bursts of 25 pulses at 8, 40, 200, and 1000 Hz had the same effect on the MP, except for the lag in the effect of the 8 Hz burst (because it took 3 s to deliver all 25 pulses at this rate). Each trace is the average of 17–22 cells. Note a different calibration for panel F.

Fig. 4(A) compares optical ∆MP in dye-loaded cells (no patch clamp) and electrical ∆MP recorded in whole-cell current clamp mode (no dye added). With the exception of a fast artifact from psEP pick-up by the patch clamp amplifier, both methods measured similar ∆MP and similar time course of repolarization. This result supports the accuracy of patch clamp measurements despite the inevitable electrical interference from high-voltage psEP stimuli.

### 3.4 Effect of psEP number and delivery rate on ∆MP

The convenience and sensitivity of optical ∆MP measurements make it a method of choice to reveal psEP bioeffects and compare different psEP treatment protocols. While a single 500-ps EP at 190 kV/cm caused only marginally significant change of membrane conductance, the respective ∆MP response was robust and reproducible (Fig. 4(A)). Thus far, the membrane depolarization by psEP can be regarded as the most sensitive biological endpoint. However, the drawbacks of optical ∆MP measurements are the lack of information on the initial MP in individual cells and the expected decrease of sensitivity as the MP approaches zero.

Indeed, increasing the number of 500-ps pulses to 5, 25, and 125 (in 1-kHz bursts) increased the response non-linearly (Fig. 4B-E). It is difficult to discern if this non-linearity resulted from a reduced membrane permeabilization effect of those psEP which were delivered fast after the first psEP in the burst; or from active physiological correction of the MP by the cell; or just from decreasing the sensitivity of the method as the MP approached zero.

Delivering a burst of 25 pulses at 1000, 200, or 40 Hz produced identical effects (Fig. 4(F)). Moreover, the effect of 8-Hz bursts, by the time when all 25 pulses were delivered, was the same as of the other pulse rates. These data are consistent with additive effects of individual pulses in the burst, concurrently with on-going restoration of the resting MP (by either membrane resealing or activation of ion pumps). The additive effect of sequential pulses is yet another psEP feature that matches earlier data for nsEP [35].

## 4 Summary

We found that even a single psEP at 190 kV/cm can cause lasting cell membrane permeabilization. The effect was enhanced by delivering multiple pulses but not by varying their frequency. Cell depolarization was measured similarly by patch clamp and by an MP-sensitive fluorescent dye, indicating that both methods are accurate. Finally, psEP-induced conductance possessed the same features as were reported earlier for nsEP, namely the maximum increase at negative MP, inward rectification, MP sensitivity, and gradual recovery over tens of seconds. We therefore infer that the nature and physical properties of psEP-opened pores are not different from those opened by nsEP, despite qualitative difference in the membrane charging mechanisms (see [17] for discussion).

The only notable difference from nsEP effects was profoundly weaker membrane permeabilization in CHO cells as compared to either NG108 or RHN. While we do not know its cause, this difference is helpful to explain the lack of Ca^2+^ mobilization by psEP in CHO cells [17]. In cells which express VGCC, even a modest membrane permeabilization led to depolarization, VGCC opening, and Ca^2+^ activation. Indeed, blockage of VGCC inhibited psEP-induced Ca^2+^ activation, proving that Ca^2+^ entry through electropores at tested psEP parameters was non-detectable. In CHO cells, the lack of endogenous VGCC expression coupled with 4–5-fold weaker membrane permeabilization makes a good explanation why no Ca^2+^ response was detected. One may expect that psEP stronger than 190 kV/cm will evoke Ca^2+^ transients in CHO cells just as well as relatively low-amplitude nsEP [18], [19], but this conjecture can be tested only when the proper psEP generation and delivery technology is developed.
